# Green Extraction of Bioactive Compounds from Marine Macroalgae: Chemistry, Pharmacological Activities, and Biotechnological Applications

**DOI:** 10.3390/md24060198

**Published:** 2026-06-04

**Authors:** Yongjing Guan, Yuxin Guo, Luoxuan Lin, Lizhu Zhang, Weichao Chen, Chao Zhao

**Affiliations:** 1 State Key Laboratory of Mariculture Breeding, College of Food Science, Fujian Agriculture and Forestry University, Fuzhou 350002, China; 2 School of Agriculture and Biology, Shanghai Jiao Tong University, Shanghai 200240, China

**Keywords:** marine macroalgae, bioactive compounds, marine natural products, pharmaceutical applications, marine biotechnology

## Abstract

Marine macroalgae are widely distributed renewable resources that offer substantial economic and environmental benefits. This review comprehensively examines seaweeds from the phyla Chlorophyta, Heterokontophyta, and Rhodophyta, highlighting key advances and persistent challenges. Global seaweed production is highly concentrated: Asia accounts for 97% of the total, with China as the dominant producer. These seaweeds synthesize a diverse array of bioactive compounds, including sulfated polysaccharides, phlorotannins, terpenoids, proteins, peptides, polyunsaturated fatty acids, and pigments. Notably, brown algae represent the richest source of both phlorotannins and polyunsaturated fatty acids. To recover these valuable compounds efficiently, a range of advanced green extraction techniques have been developed, such as enzyme-assisted, microwave-assisted, ultrasound-assisted, and supercritical fluid extraction, along with natural deep eutectic solvents. These methods consistently outperform conventional approaches in terms of yield, extraction time, and environmental sustainability. The isolated compounds exhibit a broad spectrum of validated pharmacological activities, including immunomodulatory, anti-inflammatory, anti-diabetic, neuroprotective, antitumor, and antiviral effects. Consequently, they have found diverse applications in functional foods, biomedicine, cosmetics, agriculture, aquaculture, and environmental protection. Despite this promise, critical challenges remain in elucidating structure–activity relationships, developing scalable and sustainable extraction protocols, and advancing clinical translation. Future research should prioritize the discovery of novel marine bioactives, the enzymatic production of oligosaccharides, efficient purification of algal proteins and peptides, and the scaling-up of industrial processes to fully realize the pharmaceutical and biotechnological potential of marine macroalgae.

## 1. Introduction

Seaweed, also known as macroalgae, refers to macroscopic, multicellular marine algae primarily belonging to the phyla Chlorophyta (green algae), Heterokontophyta (brown algae, class Phaeophyceae; formerly classified as Phaeophyta), and Rhodophyta (red algae) [1,2]. Chlorophyta primarily contain chlorophyll a and b, whereas Heterokontophyta are characterized by the presence of fucoxanthin, and Rhodophyta contain phycobilins (phycoerythrin and phycocyanin) as their main accessory pigments [3]. These photosynthetic, non-vascular, eukaryotic organisms are widely distributed in coastal regions from the tropics to the polar zones and serve as important renewable resources that contribute substantially to the structure and function of marine ecosystems [4,5]. Seaweeds exhibit a wide range of sizes, from filaments only a few millimeters in length to large thalli reaching up to 60 m [6]. Compared with terrestrial plants, marine algae present economic and environmental benefits, including rapid growth rates, cultivation in saltwater without the need for fertilizers, and no competition for arable land or freshwater resources. Consequently, they have been described as “promising plants of the millennium” [7].

Green algae are divided into two major lineages: Chlorophyta, which encompasses most green algae, and Streptophyta, which includes charophytes and land plants. As algae evolved, researchers divided them into six groups: Prasinophyceae, Ulvophyceae, Trebouxiophyceae, Chlorophyceae, Chlorodendrophyceae and Pedinophyceae [8]. Currently, there are over 7700 species of green algae worldwide, predominantly inhabiting freshwater, marine, and wetland environments (http://www.algaebase.org/) [9]. Among the green macroalgae, *Ulva* (commonly known as “sea lettuce”) is one of the most abundant genera globally, mainly distributed in temperate and subtropical oceans and containing diverse bioactive components [10]. *Ulva prolifera*, the primary algae causing green tides, threatens marine ecosystems but is also utilized in traditional medicine and functional foods [11].

Brown algae are highly evolved multicellular algae, with approximately 2144 known species (http://www.algaebase.org/). They are mostly found in the cold coastal waters of mainland waters and are uncommon in freshwater, reaching lengths of up to 45 m. The primary species of brown seaweed encompass *Sargassum*, *Ascophyllum*, *Undaria* and *Laminaria* [12]. Among these, *Sargassum* is recognized as one of the largest genera of brown algae. Due to its rich composition of polysaccharides, proteins, polyphenols, flavonoids, sterols, carotenoids and other bioactive compounds, *Sargassum* has been called the “medicinal food of the 21st century” [13,14], showing a variety of pharmacological properties. *Laminaria*, characterized by its brown hue and potential to grow up to 4 m in length, has been referred to as a “longevity food”, a “sea vegetable” and an “iodine champion”. Additionally, it serves as a significant source for the production of dietary fiber [15]. *Laminaria* is cultivated as a key seaweed species for the economy in Korea, Japan and China [16].

Red algae are considered the oldest large eukaryotic algae found in both freshwater and marine environments, comprising around 7586 species (http://www.algaebase.org/). In cold and temperate regions, most algae prefer the intertidal and subtidal zones of rocky coasts [17]. Compounds found in red seaweed include polysaccharides like carrageenan or AGAR, proteins, amino acids, sterols, carotenoids, bromophenol, and other naturally bioactive substances [18]. Common red algae include *Rhododerma palmatum*, *Porphyra*, *Carrageum*, and *Gelidium amansli*. *Gracilaria* is considered an economic resource because of its ability to achieve high-yielding biomass with commercial value. It is a crucial provider of agar [19,20]. *Porphyra* is a kind of edible algae; it is rich in protein and is not only nutritious, but also has a delicious taste.

This review aims to comprehensively examine the green extraction, chemical diversity, pharmacological activities, and biotechnological applications of bioactive compounds derived from marine macroalgae. To this end, we conducted an electronic search of the PubMed and Web of Science databases covering English literature published from 2000 to 2025 in this review. The search strategy utilized the following keywords: macroalgae, seaweed, Chlorophyta, Phaeophyta, Rhodophyta, bioactive compounds, polysaccharides, phlorotannins, terpenoids, fatty acids, proteins, peptides, green extraction, enzyme-assisted extraction, microwave-assisted extraction, ultrasound-assisted extraction, supercritical fluid extraction, pharmacological activity, immunomodulatory, anti-inflammatory, antidiabetic, neuroprotective, antitumor, antiviral, functional food, cosmeceutical, agriculture, and bioremediation. Various combinations of these search terms were employed to comprehensively retrieve relevant literature. Further screening was performed by reviewing the titles and abstracts of the identified articles. Articles that did not focus on the bioactive compounds, extraction technologies, pharmacological activities, or applications of marine macroalgae were excluded. The majority of the selected literature was published between 2018 and 2025.

## 2. Global Production and Distribution of Seaweed

Algal resources are highly abundant worldwide, with nearly 10,000 species recorded to date, of which more than 100 species are currently under economic utilization. Japanese kelp (*Saccharina japonica*), Eucheuma seaweeds nei (*Eucheuma* spp.), and *Gracilaria* seaweeds (*Gracilaria* spp.) together account for 35.5%, 23.2%, and 14.8% of global seaweed production, respectively. The majority of algae resources consist of brown and red algae, with green algae production being comparatively low. Over the last few decades, the production of algae has experienced significant growth, as evidenced by the nearly threefold increase in global seaweed production, which rose from 12 million tons in 2000 to 38 million tons (wet weight) in 2022. Overall, 97% of this comes from aquaculture, mostly mariculture, and 3% from wild harvesting [21].

**Figure 1:**
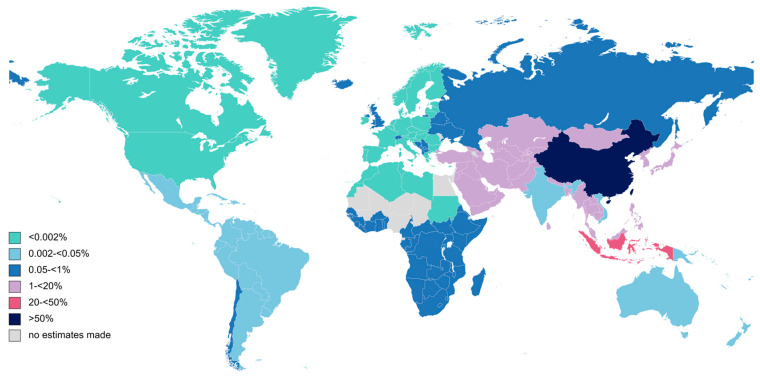
The distribution of global seaweed production in the year 2022 [21].

Seaweed production globally is primarily sourced from five continents, with Asia contributing 97% (Figure 1) [21]. In Asia, seaweed cultivation accounts for 99.30% of the total seaweed output. China is the top producer globally, contributing 60% of the world’s seaweed, with Indonesia following at 25%. Excluding Nigeria, Africa contributed just 0.5% to global seaweed production, with most of it found in Sub-Saharan Africa. America, Europe and Oceania had less seaweed production, accounting for 0.06%, 0.08% and 0.03% respectively (Figure 1). Therefore, if the Asian seaweed resources are fully utilized and developed, it will be a huge treasure house.

## 3. Chemical Composition Diversity of Seaweed Bioactive Compounds

Seaweed is valuable for applications in food, cosmetics, biomedicine and other areas. The chemical composition of seaweed is relatively complex, and its chemical structure types include carbohydrates, proteins, lipids, terpenoids and so on. The amount of these chemicals varies by season and region, and varies by species of seaweed.

Phenolic compounds are the most structurally variable and abundant group of metabolites in seaweeds [22]. The most studied seaweed polyphenols are the phlorotannins, found only in marine brown seaweeds. Among them, Laminariaceae is identified as the richest source of phlorotannins in marine algae, functioning as primary and secondary metabolites in brown seaweeds [23]. Conversely, the predominant phenolic compounds in green and red seaweeds are bromophenols, flavonoids, phenolic acids, and phenolic terpenoids [24]. These components are considered stimulatory metabolites as they function as defensive agents against various stimuli and serve as defensive mechanisms in algae against herbivorous animals and UV radiation [25]. Phenolic compounds from seaweeds have attracted considerable interest due to their diverse biological activities, including antidiabetic, anti-inflammatory, antimicrobial, and antiviral effects [26,27]. Furthermore, seaweeds, particularly red algae, are rich sources of structurally diverse terpenoids [28]. Within the seaweeds, the family Rhodomelaceae is widely regarded as a terpenoid reservoir owing to its wide chemical diversity and architecturally diverse terpenoids [23].

Seaweeds contain a small volume of fatty acids, about 1–5%, and their polyunsaturated fatty acid content is comparable to or even exceeds that of vegetables that grow in soil on land, which has important influences on human well-being [29]. The main omega-3 unsaturated fatty acids that exist in seaweeds are α–linolenic acid (ALA, 18:3n–3), eicosapentaenoic acid (EPA, 20:5n–3) and docosahexaenoic acid (DHA, 22:6n–3) [30]. Among them, EPA and DHA are widely acknowledged as the two core polyunsaturated fatty acids in marine lipids that minimize the risk of cardiovascular afflictions [31]. In addition, myristic (C14:0) and palmitic (C16:0) acids are the most abundant saturated fatty acids [30]. Japanese and Korean red algae contain more dodecenoic acid, and wakame, as well as kelp, embrace more arachidonic acid [32,33]. Green seaweeds typically contain higher levels of unsaturated fatty acids than saturated fatty acids. Palmitic acid (C16:0) is the predominant saturated fatty acid, while unsaturated fatty acids include oleic (C18:1), linoleic (C18:2), α–linolenic (C18:3), and eicosapentaenoic (EPA, C20:5) acids [34]. Brown algae have the highest fatty acid content relative to green and red algae, with more than 50% of the fatty acids available in the form of EPA.

Protein content varies significantly among seaweed groups. Green macroalgae have been reported to contain protein levels up to 55–70% of dry weight in certain species under optimal growth conditions [35], while brown algae typically show approximately 5–15% dry weight [36]. Red algae exhibit intermediate values, with some species such as *Porphyra* and *Pyropia* reaching up to 24% protein [37]. The difference in protein content is predominantly impacted by the biological kinds and growth environment of algae [38]. Broadly speaking, protein content exhibits a high concentration in spring and winter, while relatively low in summer [39]. In addition, algae possess an elevated nutritive value of protein, as they contain large amounts of every single essential amino acid, in particular, acidic residual aspartic acid and glutamic acid [40]. Furthermore, due to their protein richness, algae are a good source of bioactive peptides with likely applications in the food, pharmaceutical and cosmetic sectors [41]. Marine algae produce bioactive nitrogen-containing compounds, including lectins, phycobilin, and alkaloids. Algal lectins exhibit immunomodulatory activity; phycobilins show antioxidant, antitumor, and hepatoprotective effects [3]; and halogenated alkaloids from algae display significant bioactivity in lipid metabolism [42].

Seaweed is a dependable source of naturally occurring volatile compounds, for instance, sulfur-containing compounds and halogens [43]. Cell walls of most marine red and brown algae are abundant in sulfurated galactan polymers (carrageenan, AGAR, fucose gum) [44]. In addition, algae contain a variety of volatile odor substances, such as *Enteromorpha* containing dimethyl sulfur, kelp containing methyl mercaptan, and seaweed containing dimethyl sulfur parent (dimethyl–β–propionate). Volatile halogenated compounds, for example, bromophenol, are prevalent marine secondary metabolites. Studies have shown that natural bromophenol bis (2,3,6–tribromo–4, 5–Dihydroxybenzyl) ether is found in marine red algae. It can protect HaCaT cells from oxidative damage through the Nrf2–mediated pathway, which is a promising antioxidant [45,46].

Green algae polysaccharide, commonly referred to as ulvan, contains up to 65% (dry weight) polysaccharides and is primarily made up of rhamnose, xylose, sulfate groups, glucuronic acid, galactose and arabinose [47,48]. The sulfate content in *Ulva lucida* is correlated with antioxidant activity. It was reported that higher levels of sulfate exhibited higher antioxidant activity [49]. The most common polysaccharides derived from brown seaweeds include fucoidan, alginate, and laminarin, along with the sugar alcohol mannitol [50]. Rockweed polysaccharides represent sulfated polysaccharides of brown algae, and they constitute 10–20% of seaweed dry weight percentage and are found to have anticoagulant, antiviral and anticancer properties in wakame, *Laminaria* and *Sargassum cinereum* [51,52,53,54]. Alginate serves as a critical constituent of the cell wall and a soluble dietary fiber that helps to lower blood cholesterol and glucose levels [55,56]. Mannitol, a sugar alcohol produced by photosynthesis, accounts for 20–30% of the total dry weight of brown algae and is present in *Laminaria* and *Ecklonia*, among others, and to a minor degree in green microalgae [57,58]. It is widely used in cosmetics and pharmaceuticals for its moisturizing and antioxidant properties [59]. Red algae polysaccharides capture 40–50% of their dry weight, and agar and carrageenan serve as the most common cell wall polysaccharides in red algae [60]. Carrageenan is the largest-selling algal hydrocolloid, with a market value approximately three times that of agar and substantially greater than alginate [61]. Agar, formed from different units of galactose, has gel properties and is extensively utilized as an algal gum [62].

## 4. Green Extraction Technologies for Marine Algal Bioactives

**Figure 2:**
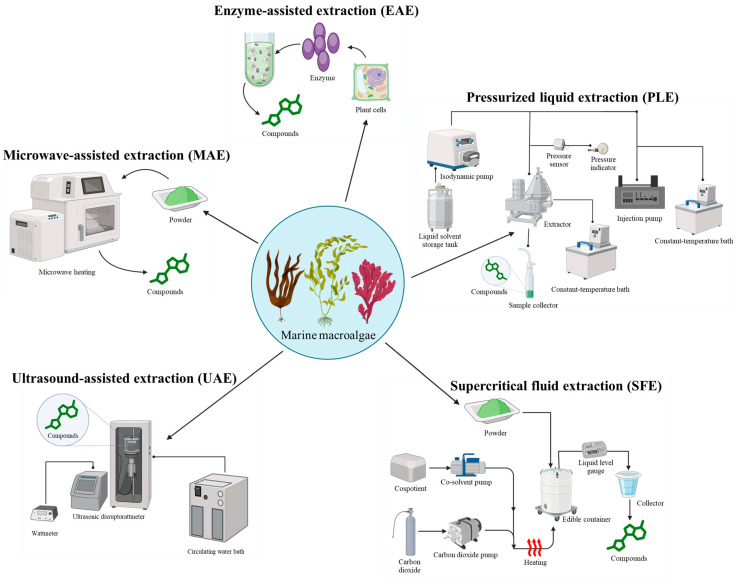
Green extraction technologies of seaweed-derived bioactive compounds.

Seaweed is a rich source of bioactive compounds with diverse biological functions, including polysaccharides, proteins, and omega–3 polyunsaturated fatty acids. The biological activity potential of fatty acids, carotenoids, vitamins, minerals, and different algae has been extensively explored in the literature [63]. Bioactive compounds are hypersensitive to extraction techniques on the basis of heating or solvent use. Additionally, these technologies are lengthy and labor-intensive. It is vital to determine and innovate new and productive extraction processes to make use of the bioactive substances in algae. For this purpose, algae researchers have been committed to developing new technologies that are more resource-efficient in terms of yield, time and cost. Enzyme-assisted extraction (EAE), microwave-assisted extraction (MAE), ultrasonic-assisted extraction (UAE), supercritical fluid extraction (SFE), pressurized liquid extraction (PLE) and other advanced extraction technologies have been successfully implemented in the disciplines of food science and pharmaceuticals to extract algal bioactive compounds (Figure 2).

For algal polysaccharides, to illustrate, fucoidan, phytoran, in addition to furcellaran, these polysaccharides are generally extracted with acid or water as solvent, and then subsequently precipitated with calcium chloride to separate alginate [64]. Phenolic compounds in algae are usually extracted by direct immersion with water or organic compounds such as methanol, ethanol, etc. Studies have found that 70% of acetone is more effective than water in extracting polyphenols. Generally, the subsequent purification of the extract is carried out by high-performance liquid chromatography (HPLC) or silica gel chromatography, so that the purity of the obtained polyphenols is higher [65]. With respect to ω–3 fatty acids, especially DHA and EPA, they are usually extracted by chloroform solvent extraction and then analyzed by gas chromatography (GC) or thin-layer chromatography [66]. Natural Deep Eutectic Solvents (NADES) are composed of natural metabolites and exhibit melting points lower than those of their individual constituents, thereby providing advantages such as biodegradability, non-toxicity, and cost-effectiveness [67]. Notably, NADES have been effectively utilized for the extraction of polyphenols, particularly phlorotannins, from brown seaweeds, resulting in higher yields and enhanced antioxidant activity compared to conventional solvents like methanol or ethanol [68,69]. A significant advantage of NADES lies in their capacity to simultaneously extract both hydrophilic and lipophilic compounds. For example, research on the brown seaweed *Fucus vesiculosus* has shown that NADES can concurrently extract hydrophilic ascorbic acid and polyphenols along with lipophilic astaxanthin in a single process [68]. This dual polarity, which can be adjusted by modifying the component ratios, obviates the need for separate solvent systems, thereby reducing processing time and environmental impact [70].

In recent years, supercritical fluid extraction, ultrasound and other extraction technologies have been universally deployed in the extraction of fatty acids [71]. Obluchinskaya et al. compared dynamic maceration (DM) and ultrasound-assisted extraction (UAE) for fucoidan from four Arctic brown algae, finding that UAE enhanced fucoidan yield by 43.2% and uronic acid content by 22.0%, but decreased phlorotannin content by 53.7% [72]. In contrast, DM–extracted fucoidans exhibited higher antioxidant activity and superior anticancer effects against HeLa G–63 cervical cancer cells due to preserved phlorotannins [72]. Algae also contain many bioactive peptides and proteins. So far, the extraction and separation of proteins, peptides and amino acids from macroalgae are primarily carried out on a laboratory scale, usually using polysaccharide enzyme-assisted extraction, water extraction and alkali extraction [73]. Chromatography technology is gradually being widely used for the separation and purification of bioactive proteins, peptides and amino acids. In addition, spectrophotometric methods, such as liquid chromatography-mass spectrometry (LC–MS), ultrafiltration and gel permeation chromatography, have recently been used to separate polypeptides from protein sources into parts with contrasting molecular weights and structures [74]. Traditionally, carotenoids in algae are collected by solvent extraction with hexane as a nonpolar solvent. However, new technologies for efficient extraction have been developed recently, such as direct extraction of vegetable oil, supercritical fluid extraction and pressurized liquid extraction. These methods improve the extraction efficiency of carotenoids. At the same time, the solvent in these new technologies can usually be well separated from carotenoids, especially in supercritical fluid technology. Because of the use of pressurized liquid–gas extraction, it will automatically volatilize during the separation process. It will not remain in the extract, thereby enhancing the purity of the extracted substance [75].

Each extraction technology presents distinct advantages and limitations. Conventional methods, such as solvent extraction with organic solvents (e.g., hexane, chloroform, methanol), are simple, cost-effective, and well-established, but they are time-consuming, require large solvent volumes, and may leave toxic residues, raising environmental and safety concerns. Additionally, heat-based techniques risk degrading thermolabile compounds like polyphenols and polyunsaturated fatty acids. In contrast, advanced green technologies offer significant improvements: EAE operates under mild conditions, preserving bioactivity, though enzyme costs and strict parameter control limit scalability; MAE and UAE drastically reduce extraction time and solvent use, but localized heating or cavitation may occasionally damage sensitive compounds; SFE produces solvent-free, high-purity extracts ideal for lipophilic compounds like carotenoids, yet high equipment costs and poor efficiency for polar compounds restrict its application; and PLE enables rapid extraction with minimal solvent but may degrade heat-sensitive bioactive at elevated temperatures. NADES technology integrates the principles of green chemistry with high extraction efficiency for both polar and moderately polar compounds, offering the distinct advantage of simultaneous recovery of hydrophilic and lipophilic compounds, although challenges related to viscosity persist. Therefore, the selection of an appropriate extraction method must balance yield, purity, cost, and compound stability, with hybrid approaches increasingly recommended for comprehensive bioactive recovery.

## 5. Pharmacological and Biological Activities of Seaweed-Derived Compounds

### 5.1. Immunomodulatory Effects of Seaweed Bioactives

**Figure 3:**
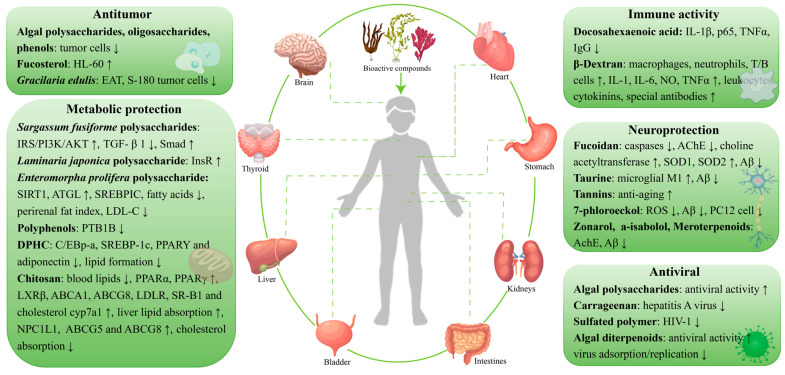
Functional properties of seaweed-derived bioactive compounds [76,77,78,79,80,81,82,83,84,85,86,87,88,89,90,91,92,93,94,95,96,97,98,99,100,101,102,103,104,105,106,107,108,109,110,111,112,113,114,115,116,117,118,119,120,121,122,123,124,125,126,127,128,129,130,131,132,133,134,135,136,137,138]. Upward arrows (↑) indicate an increase or upregulation, and downward arrows (↓) indicate a decrease or downregulation of the corresponding parameters.

The immune system in vertebrates acts as immune surveillance, defense and regulation. This system comprises immune organs (spleen, lymph nodes, thymus, etc.), immune cells (lymphocytes, mononuclear phagocytes, neutrophils, etc.), and immunologically active substances (cytokines, lysozyme, immunoglobulins, and complement proteins) [76]. When pathogenic microorganisms such as bacteria and viruses enter the human body, the innate immune system initially recognizes pathogen-associated molecular patterns through pattern recognition receptors. This triggers a cascade of immune responses, including the activation of phagocytic cells that engulf and destroy extracellular pathogens, cytotoxic CD8^+^ T cells and natural killer cells that eliminate infected host cells, and the subsequent activation of the adaptive immune system, which produces specific antibodies. These antibodies bind to the pathogens, neutralizing them and marking them for elimination by other immune cells [77]. In addition, the immune system is also related to aging and cancer cell clearance. Maintaining the normal operation of the immune system promotes the normal metabolism of the body. With the progress of science and technology and the sustainable refinement of people’s living standards, more and more attention has been paid to the immunomodulators used to improve the immune function of the body. Over the last several years, natural products have been widely studied by scholars given their rich sources, wide distribution, rich biological activities, and low toxicity and side effects. The development of natural products with immunomodulatory activity is of great significance for raising the immune function of the body and preventing and treating immune-related disorders, including immunodeficiencies, autoimmune diseases, and chronic inflammatory conditions. Studies have shown that natural bioactive compounds with immunomodulatory effects mainly include polysaccharides, proteins, peptides, flavonoids, saponins and volatile oils [78]. Therefore, algae, which are rich in many active substances, are also used in the development of natural immune regulators (Figure 3).

The current research on active substances of algae shows that algal components can be involved in a wide variety of immunoregulatory activities through immune organs, immune cells, immune factors and other aspects, such as promoting macrophage IL–1, IL–6, NO, and TNF–α. The production of inflammatory mediators can improve their phagocytosis of bacteria and viruses, improve the activity of macrophages and T/B cells, and regulate the MAPK/NF–κB signal pathway, which regulates the release of cytokines, etc. [79]. Fucoidan from *Fucus vesiculosus* (12.5–100 μg/mL, 24 h) notably increased NO production and phagocytic activity in RAW 264.7 macrophages compared to LPS (1 μg/mL) control [79]. Algal compounds also demonstrate immunomodulatory effects in vivo. Calves receiving a docosahexaenoic acid-rich algal supplement (20–30 g/day for 56 days) showed reduced *IL–1β*, *TNF–α*, and *p65* mRNA expression, with 20 g/day as the minimal effective dose [80]. Similarly, broiler chickens fed 1–2% *Ascophyllum nodosum* extract for 35 days had significantly higher CD4^+^ and CD8^+^ T cell populations in cecal tonsils (*p* < 0.05) [81]. Algal β–glucan, prevalent in algae, boosts immune activity through various mechanisms. In vitro, algal β–glucan (1–100 μg/mL, 48 h) increased IL–6, TNF–α, and ROS production in human macrophages, peaking at 50 μg/mL [82]. In vivo, oral algal β–glucan (50 mg/kg daily for 7 days) enhanced splenocyte proliferation and NK cell activity in mice [83].

The anti-inflammatory properties of algal compounds have been substantiated in various studies. Fucoidans from five brown seaweed species could mediate anti-inflammatory effects via inhibition of protein denaturation and stabilization of human red blood corpuscle (HRBC) membranes. The protein denaturation inhibition was concentration-dependent and strongly correlated with fucose content, while moderately correlated with sulfate content. Notably, purified fucoidan (FV2) exhibited the most promising activity (IC_50_ = 0.20 mg/mL), outperforming the reference drug diclofenac sodium (IC_50_ = 0.37 mg/mL) [84]. Specifically, fucoidan derived from brown seaweeds, at concentrations ranging from 25 to 200 μg/mL, has been shown to inhibit NO, prostaglandin E2, and pro-inflammatory cytokines such as tumor TNF–α, IL–1β, and IL–6 in lipopolysaccharide (LPS)–stimulated RAW 264.7 macrophages, with an inhibitory concentration 50 (IC_50_) of approximately 75 μg/mL for NO inhibition [85]. Furthermore, topical administration has emerged as an effective delivery route: a fucoidan-based cream formulation dose-dependently inhibited carrageenan-induced paw edema and ameliorated mechanical allodynia in rats, with the efficacy at a high dose being comparable to that of diclofenac gel [86]. In a carrageenan-induced paw edema model in mice, oral administration of fucoidan at doses of 50 to 200 mg/kg resulted in a reduction in swelling by up to 62% at 4 h, which is comparable to the effects observed with indomethacin at a dose of 10 mg/kg [87]. Clinically, the application of a 2% fucoidan-based cream twice daily for eight weeks led to a significant reduction in the Eczema Area and Severity Index scores by 45.6% and pruritus scores by 52.3% in patients with atopic dermatitis (*n* = 30, *p* < 0.01 compared to placebo) [88].

At present, owing to the absence of accurate and adequate evidence in vivo for seaweed-related components, the application bottleneck of uncertain structure-activity relationship and unclear utility mechanism, the current application scope is not wide enough. As for the specific molecular mechanism of algae regulating the immune system of the body, more comprehensive experiments are needed for in-depth exploration.

### 5.2. Regulation of Glucose and Lipid Metabolism by Seaweed Compounds

Glucose and lipid metabolism disorders are epidemic chronic diseases distinguished by glucose and lipid metabolism disorders, including glucose metabolism disorder and lipid metabolism disorder. The blood glucose concentration continues to rise due to insufficient insulin secretion or insulin resistance, which leads to continuous glucose disorder and eventually diabetes. Diabetes causes some chronic diseases, mainly including damage and pathological changes in the kidneys, eyes, heart, blood vessels and other tissues [89]. The normal metabolism of lipids exerts a key function in maintaining the dynamic balance of energy metabolism, promoting tissue regeneration, maintaining the stability of the cell membrane and providing trace elements for the body. Abnormal operation of lipid metabolism will lead to abnormal body fat composition or lipid deposition. Abnormal lipid metabolism is closely related to gender, growth time, diet, exercise frequency, genetics and other factors [90,91,92].

Possible mechanisms include inflammatory factors, oxidative stress, etc. Simultaneously, any abnormality in multiple tissues and organs in the body may cause lipid metabolism disorder. Disordered lipid metabolism can induce coronary heart disease, myocardial infarction, diabetes, arteriosclerosis, renal failure, stroke and other diseases, posing a threat to human well-being. For a series of reasons, maintaining the balance of glucose and lipid metabolism has become an important measure to maintain the normal metabolism of the body and prevent various chronic and metabolic diseases. At present, drugs for diabetes mainly reduce blood sugar in a short time by promoting glycogen synthesis and insulin secretion, which cannot achieve the goal of eradication, and long-term use will lead to liver injury and other toxic side effects. Patients with severe dyslipidemia usually need drugs to reduce cholesterol or triglycerides, but these drugs will also have more adverse reactions.

Recent studies have shown that many components of seaweed have the ability to treat glycolipid metabolism disorders (Figure 3). *Sargassum fusiforme* polysaccharides can activate the IRS/PI3K/AKT signal pathway in the glycolipid metabolism pathway, affect the expression of *HMGCR* and *SREBP–lc* genes to prevent liver fat accumulation, and inhibit TGF– β1/activation of Smad signaling pathway to delay and prevent kidney damage in diabetes [93,94]. Laminaria japonica polysaccharide can increase the expression profile of InsR protein in the liver and pancreas of diabetic mice and achieve a hypoglycemic effect [5]. *Enteromorpha prolifera* polysaccharide can regulate the mRNA levels of *InSR*, *GCK*, *APN* and *GLUT4* genes in liver and adipose tissue, slow down pancreatitis and cell apoptosis in diabetic mice, and found that it can regulate the glucose metabolism function of diabetic rats [95]. In addition, *Enteromorpha prolifera* polysaccharide can also stimulate the activity of acetyl–CoA carboxylase a2 in some organs, improve the expression of *SIRT1* and *ATGL*, promote the phosphorylation of acetyl–CoA carboxylase 1, reduce the expression of *SREBPIC* and fatty acids in adipocytes, and reduce the perirenal fat index and low-density lipoprotein cholesterol concentration [96]. PTB1B has been proven to be a functionally relevant target for the treatment of type 2 diabetes, and a variety of polyphenols extracted from *Grateloupia elliptica* have been reported to be able to effectively inhibit PTB1B [97]. Furthermore, fucoidan derived from *Fucus vesiculosus* demonstrates effective inhibition of the DPP–IV enzyme, with an IC_50_ value of 1.11 μg/mL, thereby contributing to its anti-hyperglycemic properties [98]. CRP and APN levels are associated with metabolic disorders and inflammation, and it has been reported that seaweed fiber has beneficial effects on CRP, APN and diabetes markers [99]. Diphrorethoxycarbamol (DPHC), one of the extracts of brown algae *Ishige okamura*, can reduce the level of specific proteins generated by fat, including C/EBP–α, SREBP–1c, PPARγ and adiponectin, and inhibit the lipid accumulation induced by 3T3–L1 preadipocytes in the process of lipid formation [100]. In addition, DPHC can also regulate the expression levels of *Bax*, *Bcl–2*, *caspase–9*, *caspase–3* and *PARP* in adipocytes, and apoptosis of 3T3–L1 preadipocytes is induced through the intrinsic pathway [101]. Chitosan extracted from brown algae also showed the effect of lowering blood lipids [102], and this kind of brown algal chitosan significantly increased PPARα and PPARγ levels. The expression of LXRβ, ABCA1, ABCG8, low-density lipoprotein receptor, SR–B1 and cholesterol *cyp7a1* improves liver lipid absorption. This chitosan also reduces cholesterol absorption by activating NPC1L1, ABCG5 and ABCG8 in the small intestine. Studies have shown that the bioactive compounds of seaweed can effectively regulate disorders of glucose and lipid metabolism, and therefore hold considerable promise as complementary or, in specific cases, alternative strategies to conventional pharmacotherapy.

### 5.3. Neuroprotective Properties of Seaweed-Derived Molecules

Neurodegenerative diseases are dysfunctions that occur as a result of the erosion of neurons and/or their myelin sheaths, which aggravate temporally. Neurodegenerative diseases, including Alzheimer’s disease, Parkinson’s disease, Huntington’s disease, and amyotrophic lateral sclerosis, are characterized by progressive neuronal loss and are caused by a combination of genetic mutations and environmental stresses associated with individual aging, which ultimately lead to protein aggregation [103]. Amyotrophic lateral sclerosis (ALS) is the most prevalent motor neuron disease, characterized by the progressive decline of upper and lower motor neurons, a process that eventually leads to respiratory failure and death [104,105]. Frontotemporal lobe dementia (FTD) is a dementia syndrome characterized by progressive degradation of behavior, executive dysfunction and speech impairment [106]. ALS and FTD are incurable neurodegenerative disorders with substantial clinical, genetic, and pathological overlap. Approximately 15% of ALS patients also develop FTD, and both conditions share common molecular mechanisms, including TDP–43 proteinopathy and C9orf72 repeat expansions, positioning them as two ends of a disease spectrum rather than entirely separate entities [107,108]. The World Health Organization (WHO) forecasts that neurodegenerative diseases will exceed cancer as the second most deadly disease in humans by 2040 [109]. There are no functional treatments for these diseases to date, making it urgent to understand the pathogenesis of these diseases. Currently, only a few drugs are available to slow the progression of some neurodegenerative diseases, but there is no clear cure. Neurodegenerative diseases are often associated with the production and accumulation of amyloid β (Aβ) in the body, which induces tissue oxidation and is cytotoxic, with large accumulations triggering apoptosis [4].

Recent research has indicated that many substances in seaweed have certain neuroprotective functions, as well as slowing down the effects of aging (Figure 3). Fucoidan was found to inhibit caspases, reduce AChE activity and enhance choline acetyltransferase activity, while increasing the expression of *SOD1* and *SOD2* in the CA1 region, exerting antioxidant effects and reducing the accumulation of Aβ to protect neural cells [110]. Taurine, which is rich in red algae, inhibits the activity of the pro-inflammatory substance microglial M1, which has a protective effect on neurons. High concentrations of homotaurine were identified in the green alga *Ulva lactuca*, which ties to Lys16, Lys28 and Asp23 of AB42 and reduces Aβ aggregation [111]. Tannins extracted from brown algae also have anti-aging effects, and 7–phloroeckol from *Eisenia bicyclis* showed the ability to reduce intracellular ROS levels and maintain Ca^2+^ homeostasis, thereby inhibiting PC12 cell death due to Aβ accumulation, suggesting that it may be a neuroprotective agent [112]. In addition, Zonarol extracted from the brown alga *Dictyopteris undulata*, *α*–bisabolol from *Padina gymnospora*, and meroterpenoids obtained from *Sargassum* were found to be effective in inhibiting AChE activity and delaying Aβ–induced neurotoxicity [113,114,115], suggesting that seaweeds are of great value in the treatment of neurodegenerative diseases.

### 5.4. Antitumor Activities of Algal Bioactive Substances

A tumor refers to a new growth formed by local tissue cell proliferation under the activity of diverse tumor-causing factors. Tumors can be classified into benign tumors and malignant tumors [116]. Among them, liver cancer and pancreatic cancer are extremely malignant tumors. According to the most up-to-date global cancer burden estimates provided by the International Agency for Cancer Research (IARC) of the WHO, 19.29 million new cancer cases were reported around the world in 2020, including 4.57 million new cancer cases in China, representing 23.7% of the global total [117,118]. The incidence rate of malignant tumors is increasing globally, posing a serious threat to human health. Radiotherapy, which employs high-energy radiation directed at the tumor site, has become one of the standard treatment modalities for locally advanced cancer due to its strong tissue penetration and effective tumor-cell killing capacity [119,120]. However, there are still major challenges in the current tumor treatment: on the one hand, most solid tumors are characterized by hypoxia, and hypoxic tumor cells are less sensitive to radiation, which can readily lead to the development of radiation resistance [121]. In addition, the high dose and high frequency radiation therapy of ionizing radiation in clinical tumor treatment caused serious damage to the normal tissues near the tumor [122]. Improving local tumor control while minimizing collateral damage to surrounding normal tissues remains a persistent challenge in radiotherapy. Hence, it is vital to establish a safer and more effective protocol to assist existing clinical cancer therapy methods and improve treatment outcomes.

The research on anti-tumor applications using algae has a history spanning several decades (Figure 3). Through the relevant research using human cancer cell lines, it has been discovered that a variety of algal extracts have anti-tumor effects. Algal extract has good anti-tumor potential. Among them, algal polysaccharides are the most widely reported. Brown algae are a common coastal food, and anti-tumor research related to brown algae is also a key research hotspot. The anti-tumor components derived from algae include sulfated polysaccharides such as carrageenan and fucoidan [54,123], algal oligosaccharides such as brown algal oligosaccharides [124], phlorotannins such as eckol and dieckol [125], as well as terpenoids, steroids, quinones, and lactones [126]. At present, there are increasing reports of seaweed anti-tumor drugs. For example, a kind of fucosterol isolated from brown algae has strong differentiation-inducing activity on human promyelocytic leukemia cell HL–60 by promoting apoptosis [127]. Research has also shown that *Gracilaria edulis* extract exhibits significant inhibitory effects on Ehrlich ascites tumors and S–180 tumor cells [128]. The development and research of seaweed anti-tumor drugs remains time-consuming and high-investment research, which requires multidisciplinary and multi-dimensional cooperation. The identification of marine antitumor drugs with potent activity and unique structural features necessitates large-scale systematic pharmacological screening, rigorous data analysis, and extensive clinical investigation. However, the collection of a large number of marine biological resources is relatively difficult, so exploring the methods and techniques of artificial cultivation of algae, using biological engineering technology to cultivate algae, or using genetic engineering technology to clone and express anti-tumor active compounds are all important options.

### 5.5. Antiviral Potential of Seaweed Extracts and Isolated Compounds

Virus infection is one of the crucial factors of body injury and infectious diseases. After the virus invades the body, the natural immune system recognizes the relevant molecular patterns of the pathogen through the pattern recognition receptor and then triggers the natural antiviral immune response to induce the production of interferon, inflammatory factors and other substances to resist the virus invasion. Many viruses pose significant threats to human health. For example, human immunodeficiency virus (HIV) and rabies virus can be life-threatening, while influenza virus and hepatitis viruses are responsible for widespread infectious diseases [129]. At present, viral diseases can usually be prevented by vaccination; however, effective vaccines are still unavailable for many serious viral diseases. Because viruses cannot survive independently, they rely on host cells to obtain energy and materials to complete their own reproduction, but also change the metabolic mode of host cells to obtain a better living environment [130]. The drugs currently used to treat viral infections are often associated with significant toxicity; for instance, ribavirin can cause hemolytic anemia, while zidovudine is associated with bone marrow suppression, and many antiretroviral agents carry risks of hepatotoxicity and mitochondrial dysfunction. These adverse effects make it difficult to safely and completely eliminate the virus. As a result, it is a high-priority imperative to observe and develop new natural antiviral substances with different mechanisms and low toxicity.

It has been estimated that approximately 9% of all biomedical compounds derived from marine sources originate from algae [131]. Since the 1980s, more and more in vitro and in vivo tests have been performed, confirming that the substances in algae have certain antiviral effects. Algal polysaccharides and algal proteins are the main antiviral research objects. Although the algal polysaccharides have a wide range of architectural complexity, most of them are negatively charged and exhibit a high sulfation level. They may pressure their antiviral activity by isolating the positively charged amino acids in the glycoprotein gp120 of the virus envelope or by strong binding with specific sulfate groups [132]. Seaweed polysaccharides usually combine effective antiviral effects and low toxicity, making them a noteworthy solution to limit viral infection in clinical practice. The antiviral effects of seaweed polysaccharides mainly include direct inactivation, inhibition of virus adsorption, inhibition of virus replication and improvement of host antiviral immune response [133]. Fucoidan from *Undaria pinnatifida* was recently shown to prevent herpes simplex virus type 1 (HSV–1) infection in retinal pigment epithelial cells and inhibit the NF–κB signaling pathway [134]. The sulfated polysaccharide lambda-carrageenan has demonstrated strong antiviral activity against the infectious pancreatic necrosis virus (IPNV) by inhibiting viral replication and enhancing innate immunity in salmonid cells, with a selectivity index exceeding 142 [135]. Through research, it was found that sulfated polymer can inhibit HIV–1 infection by firmly attaching the virus gp120 protein to the CD4 molecule on the T cell surface [136]. Coupled with algal polysaccharides and algal proteins, algal diterpenoids have also been revealed to have good antiviral activity [137]. At present, the bulk of research funding addresses HIV. At the same time, more than ten antiviral drugs from algae have been used in clinical trials. Most of the research attempts to find anti–HIV treatments using algae’s active substances. It is obvious that there is great potential to use algae as an unprecedented platform or source to monitor and fabricate innovative antiviral drugs for multiple viruses in various situations [138].

## 6. Diverse Industrial Applications of Algal Bioactive Compounds

### 6.1. Functional Food and Nutraceutical Applications

Seaweeds have been incorporated into the human diet for millennia, with archeological evidence from the Monte Verde site in southern Chile documenting the consumption of nine marine algal species approximately 14,000 years ago [139]. In Asian countries, including China, Japan, Korea, and the Philippines, seaweeds have traditionally served as a staple food source, valued for their unique umami flavor and high nutritional content. A recent tri-continental dietary survey confirmed that tradition remains a primary driver of seaweed consumption in China, where between 17 and 19 macroalgal species are regularly consumed [140]. Common traditional seaweed-based foods include seaweed salads, soups, seasoned laver snacks, and seaweed powder used as a condiment. Seaweeds are naturally low in calories while being rich in essential minerals, such as iodine, calcium, and iron, as well as vitamins A, C, E, and the B group, dietary fiber, and bioactive compounds that are rarely found in terrestrial vegetables.

Beyond their traditional culinary uses, seaweed-derived polysaccharides have become important functional food ingredients. Hydrophilic colloids, including alginate extracted from brown seaweeds and carrageenan and agar from red seaweeds, are widely employed as thickening agents, gelling agents, and emulsion stabilizers in processed foods, such as dairy products, meat products, beverages, and bakery items [141]. These polysaccharides not only improve food texture and shelf stability but also function as soluble dietary fibers that can enhance satiety, reduce postprandial energy intake, and contribute to weight management and metabolic health.

In the nutraceutical sector, several seaweed species have been formally approved as novel food raw materials or are listed in health food ingredient directories. Since 2009, multiple algal resources have been successively approved for specific applications, as summarized in Table 1. The diversity of seaweed-derived functional products, ranging from dietary fiber supplements and alginate-based satiety formulations to polyphenol-rich extracts, continues to expand the role of marine macroalgae in the functional food and nutraceutical industry.

### 6.2. Biomedical and Pharmaceutical Applications

**Figure 4:**
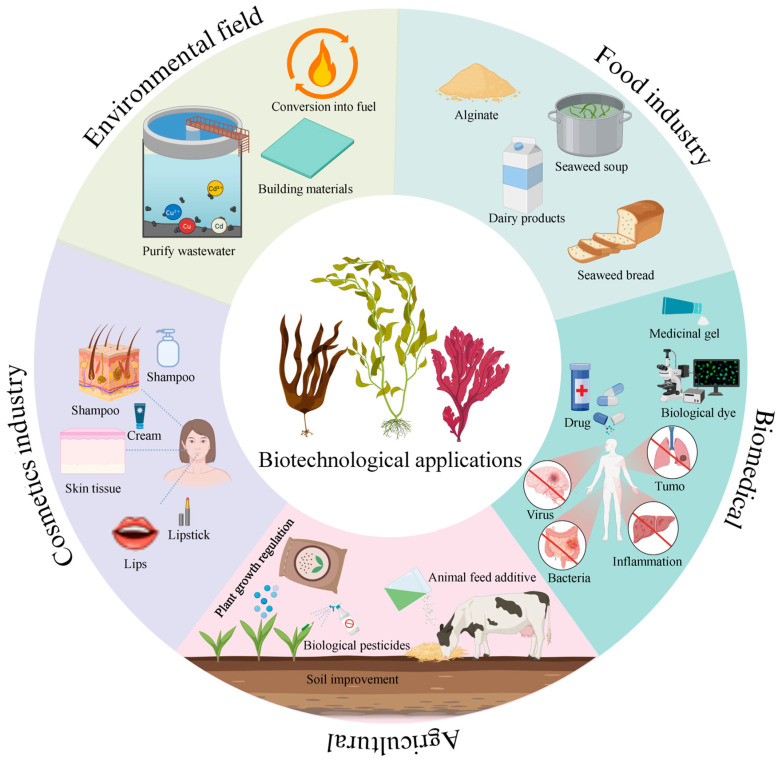
Applications of seaweed-derived bioactive compounds.

Seaweed extract is widely used in the field of biomedicine, which can be used in anti-inflammatory, antioxidant, antibacterial, antiviral and other aspects, and has a wide range of application prospects (Figure 4). In foreign countries, seaweed extract has been widely used in the preparation of antitumor drugs, immunomodulatory agents, lipid-lowering drugs, cardio-cerebrovascular disease treatment drugs and so on. For instance, polysaccharides from brown algae (such as chlorophylliane) have strong antioxidant and anti-tumor effects and have been used in the preparation of a variety of drugs, such as the anti-tumor drug alginic acid. In China, the application of seaweed extract is also expanding. Kelp oligosaccharides have been demonstrated to possess a range of biological activities, including anti-inflammatory, antibacterial, antiviral, and antitumor effects, and hold potential for the treatment of hepatitis, pneumonia, and other inflammatory and infectious diseases. In addition, seaweed extracts are also used to prepare biological dressings, medicinal gels, oral cleaning products and additional biomedical offerings [149].

Understanding the pharmacokinetics of algal compounds is crucial for assessing their therapeutic potential. Pozharitskaya et al. first characterized the pharmacokinetics and tissue distribution of fucoidan from *Fucus vesiculosus* in rats, reporting preferential accumulation in the kidneys (AUC_0_–t = 10.74 µg·h/g), spleen (6.89 µg·h/g), and liver (3.26 µg·h/g), with a mean residence time of 6.79 h. Linear pharmacokinetics were observed after topical application within the 50–150 mg/kg range [150]. Research utilizing Caco–2 cell models and mouse pharmacokinetics has shown that fucoidan oligosaccharides derived from *Kjellmaniella crassifolia*, particularly those with low molecular weights (244–1545 Da), demonstrate favorable intestinal absorption, as evidenced by apparent permeability values exceeding 1 × 10^−5^ cm/s. A correlation was observed between smaller molecular weights and more rapid absorption into the bloodstream [151]. In the context of lipid-soluble compounds, the formulation of algal docosahexaenoic acid (DHA) as a calcium fatty acid complex (DHA–Ca) in mice resulted in a 1.81–fold increase in peak plasma concentration and a 2.26–fold increase in the area under the curve (AUC_0_–∞) compared to conventional DHA. This formulation also extended the mean residence time to 29.7 h and enhanced accumulation in the liver, heart, and brain [152]. These findings underscore the significance of molecular weight and formulation strategies in enhancing the bioavailability of algal bioactives.

A significant biomedical application of algal polysaccharides is their use in agar-based hydrogels for the production of soft capsule shells. A pioneering technology was developed for preparing herbal oil extracts using rotary-pulsation extraction and formulating soft halal capsules with agar derived from *Gracilaria* species as a plant-derived alternative to animal gelatin [153]. In contrast to traditional gelatin capsules, which are constrained by issues such as drug cross-linking, moisture sensitivity, and dietary restrictions related to religious or vegetarian practices, agar-based hydrogels provide a plant-derived alternative characterized by enhanced stability and safety [154]. The properties of agar-based materials can be further tailored through the use of plasticizers; for example, the addition of glycerol, sorbitol, citric acid, or sodium citrate modulates the moisture absorption and mechanical properties of agar films, enabling their use in pharmaceutical and food packaging [155]. Recent research has focused on optimizing gel compositions for this purpose. For instance, Liu et al. [156] demonstrated that optimized κ/ι–carrageenan blends, particularly at a 5:5 ratio, exhibit superior mechanical properties and rapid disintegration across various pH environments. These formulations address the limitations of conventional capsules while promoting the broader use of marine algal resources in pharmaceutical applications.

Several algal compounds have demonstrated clinical efficacy. A randomized trial of *Spirulina platensis* peptides for periodontal wound healing (n = 20, split-mouth) showed significant reductions in plaque and bleeding indices at weeks 4 and 8 post-surgery, less gingival redness, lower pain scores at week 1, and reduced need for analgesics (*p* < 0.05) [157]. In metabolic disorders, alginate oligosaccharides (AOS3) from brown seaweeds exhibited potent analgesic and anti-inflammatory effects in gouty arthritis mouse models via Nrf2 activation, with no toxicity, suggesting clinical potential [158].

Algal pigments have been widely studied as biological dyes as well, which can be used in bioimaging, biomarkers and photosensitizers [159]. However, various types and sources of seaweed contain different bioactive compounds, so it is vital to select appropriate seaweed varieties and extraction methods based on the actual needs. In addition, more studies are necessary to explore the mechanism of action and determine the optimal dosage and route of medication.

### 6.3. Agricultural Applications: Crops, Livestock, and Aquaculture

The application of seaweed in agriculture is mainly reflected in soil improvement, plant growth regulation, biological pesticides and so on (Figure 4). As organic fertilizers, seaweed and seaweed-based composts enhance soil structure, water-holding capacity, and microbial activity. For example, waste seaweed compost combined with beneficial rhizobacteria significantly improved soil enzyme activities and promoted tomato seedling growth in coastal saline soils [160]. In crop production, seaweed extracts serve as effective biostimulants. The phytohormones (auxins, cytokinins, gibberellins), betaines, sterols, and polysaccharides present in seaweed stimulate seed germination, root development, and nutrient uptake, leading to improved plant vigor, stress tolerance, and yield. For instance, foliar application of seaweed extract in summer mung beans significantly enhanced growth parameters and yield attributes under field conditions [161]. Algae contain various antibacterial substances, including polysaccharides, polyphenols, and alginic acid, which can be used as biological pesticides for the control of plant diseases and pest management. Seaweed polysaccharides such as ulvan, carrageenan, alginate, and laminarin have been shown to elicit natural defense responses in olive trees against *Verticillium* wilt, demonstrating their potential as bio-elicitors for crop protection [162].

Beyond crop applications, seaweed and its bioactive compounds have been extensively investigated in livestock and aquaculture. In ruminant nutrition, the inclusion of the red seaweed *Asparagopsis taxiformis* in grass silage-based diets significantly reduced enteric methane emissions in dairy cows [163]. In monogastric animals, dietary supplementation with red seaweed (*Halymenia palmata*) improved growth performance, nutrient digestibility, and meat quality in broiler chickens [164]. In aquaculture, seaweed polysaccharides, particularly sulfated polysaccharides such as fucoidan, carrageenan, and ulvan, exert immunostimulatory effects in fish and shrimp. For example, dietary fucoidan from *Sargassum wightii* enhanced prophenoloxidase gene expression and increased resistance to *Vibrio parahaemolyticus* infection in black tiger shrimp [165]. Seaweed polysaccharides also show direct antiviral activity against major aquaculture pathogens: fucoidan from *S. wightii* reduced mortality from white spot syndrome virus (WSSV) in *Penaeus monodon* postlarvae by 33.71–61.65% [166]; sulfated polysaccharides from *Sargassum ilicifolium* inhibited fish Betanodavirus infection by blocking viral attachment [167]; and lambda-carrageenan suppressed infectious pancreatic necrosis virus (IPNV) in salmonid cells with a selectivity index exceeding 142 [135]. These multidimensional benefits position seaweed as a valuable resource for sustainable agricultural systems.

### 6.4. Cosmeceutical and Skincare Applications

Seaweed extract is widely used in cosmetics (Figure 4). Because they involve a diversity of bioactive compounds, including polysaccharides, proteins, polyphenols and so on, with moisturizing, antioxidant, anti-aging and other effects, they can be utilized in skin care, cosmetics, beauty products and other cosmetics. The polyphenols in seaweed have strong antioxidant capacity and can be used as antioxidants in skin care products to reduce oxidative stress and skin aging [168]. The polyphenol-rich fraction derived from *Padina boergesenii* demonstrates promising potential as a natural ultraviolet (UV) filter. It exhibits a DPPH radical scavenging activity of 54 ± 1% and provides protection to HaCaT keratinocytes against UVB-induced cytotoxicity, maintaining cell viability at 81.2 ± 0.1%. Furthermore, a sunscreen formulation incorporating this fraction attained a sun protection factor (SPF) of 20.55 [169]. Carrageenan and alginate are commonly found as water binders in products such as lotions, creams, toothpastes and shampoos to increase the moisturizing capacity of skin and hair [170]. The collagen in seaweed increases skin elasticity and reduces wrinkles and fine lines, and is often used in anti-aging products. Colorants used in cosmetic formulations, including eyeshadow, facial makeup and lipstick, are presently collected from red microalgae. In general, as a natural raw plant material, seaweed has a variety of effects and application scenarios. However, it should be noted that in the application process, reasonable collocation and use should be carried out according to the formula, usage amount, and skin type of specific products, so as to avoid adverse effects on the skin.

### 6.5. Environmental Applications: Bioremediation and Biofiltration

Seaweed has many applications in environmental protection, mainly using its adsorption, removal and transformation of harmful pollutants in water (Figure 4). As aquatic plants, algae can transform pollutants into harmless substances through biological adsorption, biological reduction–oxidation, biological phagocytosis and biological precipitation [171,172]. Researchers have found that some brown and red algae can absorb, reduce and transform heavy metals such as copper and cadmium in wastewater, so as to purify wastewater [173]. In addition, seaweed can reduce the use of wood and the pressure of deforestation by extracting cellulose from it and converting it into building materials such as cellulose boards and panels [174,175]. Seaweed can also be employed as carbon traps, which are subsequently used as fuel, and can offer an eco-friendly alternative supply of biomass to fuel production and for chemicals such as bioethanol and biobutanol [176,177].

## 7. Conclusions and Prospect

Seaweed serves as a cornerstone of marine biological resources with extremely wide varieties and large quantities. Seaweeds are abundant in active substances, namely polysaccharides, polyphenols, terpenoids, proteins, amino acids, polyunsaturated fatty acids, etc., which offer a wide variety of health effects. This review offers a comprehensive synthesis of current knowledge on seaweed, encompassing global production trends, chemical composition, extraction technologies, bioactive properties, and a wide array of industrial applications. Asia emerges as the predominant region for global seaweed production, contributing 97% of the total, with China as the foremost producer. Traditional extraction methods are increasingly being replaced by more efficient techniques, such as enzyme-assisted, microwave-assisted, and supercritical fluid extraction. The bioactive compounds derived from seaweed exhibit significant immunomodulatory, antitumor, neuroprotective, and antiviral activities, rendering them highly valuable for biomedical applications. In the food industry, seaweed serves as a functional ingredient, appreciated for its nutritional value and unique flavors. Additionally, it acts as a source of natural bioactive compounds in cosmetics, promoting skin health, and is utilized in agriculture as a biofertilizer and pesticide, as well as in environmental protection for wastewater treatment.

The primary challenge in the advancement of algae development is the effective application of its bioactive components, necessitating a comprehensive understanding of their functions and activity levels. Consequently, future foundational research on algal health foods should prioritize the discovery of novel food raw materials, as well as the isolation, purification, and identification of active functional factors. Although research on seaweed polysaccharides has garnered global attention and yielded promising outcomes, it remains insufficiently explored in depth. Despite the diverse and complex structures of seaweed polysaccharides, current studies predominantly focus on their primary structures, leaving their specific biological mechanisms largely unexplored. Traditional physical and chemical methods for preparing seaweed oligosaccharides are hampered by low yields, high energy consumption, and environmental pollution. In contrast, enzymatic methods offer enhanced efficiency, milder conditions, controllable processes, and environmental sustainability, positioning them as the most promising approach. Research on the production and development of algal proteins is relatively underdeveloped, with significant potential for advancement in large-scale production and high-quality protein separation technologies. The current efficiency of production processes is suboptimal, with extracts often containing numerous impurities. Algal bioactive peptides are predominantly obtained through traditional hydrolysis and purification techniques, which are characterized by their time-consuming, labor-intensive, and inefficient nature. Consequently, there is an urgent imperative to develop innovative technologies that are controllable, cost-effective, and stable, facilitating the transition from laboratory-scale to industrial-scale production. The extraction of non-protein amino acids from algae presents significant challenges, as these compounds are often structurally complex, difficult to synthesize chemically, present in low concentrations, and challenging to obtain directly from natural sources. Furthermore, the biological activities of these amino acids vary depending on the algal species and geographical origin, resulting in limited research in this domain. Algae are a rich source of polyunsaturated fatty acids, such as DHA and EPA, which are nearly absent in terrestrial vegetable oils, thereby rendering algae a valuable resource for extracting high-quality components. With their notable flexibility and high yield, marine algae represent a critical source of numerous bioactive compounds. Therefore, the development of more efficient methods for utilizing algal marine resources is of significant importance.

## Figures and Tables

**Table 1:** Marine macroalgae approved or traditionally consumed for food applications in China.

Seaweed	Phylum	Regulatory Status	Application	References
*Saccharina japonica* (Japanese kelp)	Heterokontophyta	Traditional food	Functional foods, alginate source, dietary supplement	[142]
*Undaria pinnatifida* (wakame)	Heterokontophyta	Traditional food	Snacks, functional foods, soup, salad	[143]
*Porphyra* spp. (nori/Laver)	Rhodophyta	Traditional food	Snack foods, seasoning, soup	[144]
*Gracilaria* spp.	Rhodophyta	Traditional food	Agar production, dietary fiber, salads	[145]
*Sargassum fusiforme* (hijiki)	Heterokontophyta	Approved food ingredient (1990)	Functional foods, dietary supplement	[146]
*Ulva* spp. (sea lettuce)	Chlorophyta	Traditional food	Soup, seasoning, salad	[147]
*Enteromorpha* spp. (green laver)	Chlorophyta	Approved food ingredient (2009)	Food ingredient, seasoning	[95]
*Caulerpa lentillifera* (sea grape)	Chlorophyta	Traditional food	Functional foods, dietary supplement	[148]

## Data Availability

Data are available on request.
